# Antioxidant defense system in the prefrontal cortex of chronically stressed rats treated with lithium

**DOI:** 10.7717/peerj.13020

**Published:** 2022-03-23

**Authors:** Ljubica Gavrilović, Nataša Popović, Vesna Stojiljković, Snežana Pejić, Ana Todorović, Predrag Vujović, Snežana B. Pajović

**Affiliations:** 1 Department of Molecular Biology and Endocrinology, “Vinča” Institute of Nuclear Sciences, National Institute of the Republic of Serbia, University of Belgrade, Belgrade, Serbia; 2 Department for Comparative Physiology and Ecophysiology, Institute for Physiology and Biochemistry, Faculty of Biology, University of Belgrade, Belgrade, Serbia

**Keywords:** Antioxidant enzymes, Prefrontal cortex, Lithium, Chronic restraint stress, Rats

## Abstract

**Background:**

This study aimed to investigate the effects of lithium treatment on gene expression and activity of the prefrontal antioxidant enzymes: copper, zinc superoxide dismutase (SOD1), manganes superoxide dismutase (SOD2), catalase (CAT), and glutathione peroxidase (GPx) in animals exposed to chronic restraint stress (CRS).

**Methods:**

The investigated parameters were quantified using real-time RT-PCR, Western blot analyses, and assays of enzyme activities.

**Results:**

We found that lithium treatment decreased gene expression of SOD2, as well as the activities of SOD1 and SOD2 in chronically stressed rats to the levels found in unstressed animals. However, lithium treatment in animals exposed to CRS increased prefrontal GPx activity to the levels found in unstressed animals.

**Conclusions:**

These findings confirm that treatment with lithium induced the modulation of prefrontal antioxidant status in chronically stressed rats. Our results may be very important in biomedical research for understanding the role of lithium in maintaining the stability of prefrontal antioxidant defense system in neuropsychiatric disorders caused by chronic stress.

## Introduction

The prefrontal cortex (PFC) is a region of the brain involved in the cognitive process of working memory, as well as in the regulation of thoughts, and emotions trough connection with other brain regions (Goldman-Rakic, 1995; Arnsten, 2009). Many studies have shown that neuropsychiatric disorders involve a working memory deficit caused by PFC dysfunction (Mattes, 1980; Weinberger, Berman & Zec, 1986; Deutch, 1993; Fibiger, 1995). In addition, it is known that the PFC is an area of the brain sensitive to chronic stress (Popović et al., 2017a). The literature data have shown that chronic stress can affect depression, Parkinson’s disease, schizophrenia, and other neuropsychiatric disorders (Schwab & Zieper, 1965; Goldman-Rakic, 1994; Mazure, 1995; Glahn et al., 2006; Shin, Rauch & Pitman, 2006). For example, bipolar disorder, as one of mood disorders, is associated with stress induced neuronal remodeling (Wood et al., 2004).

Chronic restraint stress (CRS) is a frequently used animal model which induces neuronal remodeling in the PFC (Wellman, 2001), and influences depressive-like behavior in rats (Popović et al., 2017b). Exposure of rodents to CRS results in dendritic atrophy of the PFC (Radley et al., 2004). Previous studies have demonstrated that CRS can trigger reactive oxygen species (ROS) accumulation in the frontal cortex, which can induce oxidative stress (Huang et al., 2014; Wang et al., 2014). Oxidative stress is an important factor in the pathogenesis of many neuropsychiatric disorders, including depression (Bhatt, Nagappa & Patil, 2020). The production of ROS is neutralized by antioxidant defense system, which can lead to the depletion of antioxidant molecules. Key enzymatic antioxidants are superoxide dismutases (SODs), which catalyze dismutation of the superoxide anion into hydrogen peroxide which is then further deactivated to water by catalase (CAT) and glutathione peroxidase (GPx). Earlier studies have showed that CRS modulates prefrontal antioxidant defense system (Popović et al., 2017a). Specifically, CRS increased the activity of prefrontal superoxide dismutase 1 (SOD1), superoxide dismutase 2 (SOD2) and CAT (Popović et al., 2017a), antioxidant enzymes representing the first line of antioxidant defense system. Lithium treatment has been shown to prevent and/or reverse DNA damage, free-radical formation and lipid peroxidation (Shao, Young & Wang, 2005; Andreazza et al., 2007; Machado-Vieira, Manji & Zarate, 2009). For example, it is known, that lithium is an effective drug in the rat behavior stabilization (Popović et al., 2019), and that it protects against oxidative stress-induced cell death (Cui et al., 2007). However, very little is known about prefrontal antioxidant defense system in chronically stressed rats treated with lithium. Due to oxidative stress direct involvement in depressive-like behavior, examining gene expression and antioxidant enzymes’ activity in the PFC of chronically stressed rats treated with lithium may be crucial to understanding the role of lithium in maintaining the stability of prefrontal antioxidant defense system in neuropsychiatric disorders brought about by chronic stress.

Our earlier research showed that CRS changed the activities of prefrontal antioxidant enzymes (Popović et al., 2017a). In this study we examined: gene expression and activity of the antioxidant enzymes SOD1, SOD2, CAT, and GPx in the PFC of chronically stressed rats treated with lithium.

## Materials and Methods

### Animals and a stress model

Experiments were performed on 11-week-old Wistar male rats weighing 300–350 g, “specific pathogen-free”, which are important for biomedical research. Three to four animals per cage were maintained under standard vivarium conditions in a temperature-controlled room (22 ± 1.0 °C) and 12 h/12 h light/dark cycle, with *ad libitum* access to water and food (Gavrilovic, Spasojevic & Dronjak, 2010). Twenty animals obtained from the Institute of Nuclear Sciences “Vinča” (Belgrade, Serbia) animal facility was randomly divided into two groups. Randomization was performed by assigning the random numbers from random number tables to the experimental animals. The experimental protocol was previously described in more details by Popović et al. (2019). The Ethical Committee for the care and use of laboratory animals of the Institute of Nuclear Sciences “Vinča” has approved the planned experiment (the opinion number 01/12). The animals from the first **CRS group** (*n* = 10) were exposed to chronic restraint stress. The rats from the second **CRS+Li group** (*n* = 10) were exposed to chronic restraint stress treatment with Li given each day immediately prior to daily restraint. Handling of animals was done quickly and carefully to avoid unnecessary discomfort. The rats were habituated to handling and treated as ethically as possible, according to the recommendations of the Ethical Committee of the Institute of Nuclear Sciences “Vinča”, Belgrade, Serbia, which follows the guidelines of the Serbian Society for the Use of Animals in Research and Education. The restraint stress procedure, described by Gamaro et al. (1999) was performed by placing each animal in a 25 × 7 cm plastic bottle. The CRS animals were exposed to 2 h of restraint stress daily during a fortnight, within random times during the light period of the light/dark cycle, to avoid habituation (Kim & Han, 2006). Data on lithium administration protocol were described in our previous research (Popović et al., 2019). The intraperitoneal lithium injections took place daily for a fortnight, as described in the protocol of Nonaka & Chuang (1998). The starting lithium dose of 1.5 mEq/kg administered over the course of the first two days was subsequently increased to 2.3 mEq/kg for the following 7 days. The highest dose of 3 mEq/kg was applied during the last 5 days of the treatment. This approach secured the maintenance of the plasma lithium above the minimum concentration (*i.e.*, 0.4 mM) specific for bipolar disorder treatment throughout the experiment. After the treatment, each animal was returned to the cage in which it was kept to avoid mixing the animals. Also, care was taken to minimize pain of the animals. The animals were monitored several times a day for 14 days. All animals survived after chronic stress and lithium treatment. To minimize the effects of circadian rhythms, the animals were sacrificed between 9:00 and 11:00 am, *i.e*., one day after the last treatment, following our previous protocol (Popović et al., 2019). Also, in accordance with our previous protocol, the animals were sacrificed under no-stress conditions by a rapid decapitation. The prefrontal cortex tissues were promptly dissected, frozen in liquid nitrogen and stored at −70° C until analyzed.

### Real-time RT-PCR

Data about methods of RNA isolation and cDNA synthesis were described in our previous research (Gavrilović et al., 2012). Specifically, prefrontal RNAs were isolated by using TRIZOL reagent (Invitrogen, Waltham, MA, USA). As stated in the manufacturer’s protocol, reverse transcription was carried out using Ready-To-Go You-Prime First-Strand Bead (Amersham Biosciences, Amersham, UK) and pd (N)6 Random Hexamer (Amersham Biosciences, Amersham, UK) primer. The more detailed description of the procedure was provided in our previous protocol (Gavrilović et al., 2012). Gene expression of antioxidant enzymes was performed using quantitative real-time RT-PCR method. Determination of SOD1, SOD2, CAT and GPx mRNA was performed by TaqMan PCR assays (Applied Biosystems, Waltham, MA, USA) for SOD1 (Rn00566938_m1), SOD2 (Rn00690587_g1), CAT (Rn00560930_m1) and GPx (Rn00577994_g1). The endogenous control was included in each analysis to correct for the differences in the inter-assay amplification efficiency and all transcripts were normalised to cyclophyline A (Rn00690933_m1) expression (Gavrilović et al., 2012). The relative expression of antioxidant enzymes was normalized to cyclophyline A and expressed in relation to the calibrator, *i.e.*, the control sample, following our previous protocol (Gavrilović et al., 2012).

### Western blot analysis

Tissue homogenizations in 0.05 M sodium phosphate buffer (pH 6.65) were described in our earlier research (Gavrilović et al., 2012). The protein concentration was measured by BCA method (Thermo Scientific Pierce, Waltham, MA, USA), described by Stich (1990). Determination of SOD1, SOD2, CAT and GPx was performed using Western blot analysis. Specifically, antibodies used for quantification of proteins were for SOD1 (dilution 1:2,000; SOD-101; Stressgen, Canada), for SOD2 (dilution 1:2,000; SOD-110; Stressgen, Canada), for CAT (dilution 1:2,000; Calbiochem, Germany), GPx (dilution 1:500; sc-30147 Santa CruzBiotechnology, Dallas, TX, USA) and for β-actin (dilution 1:1,000; ab8227, Abcam, USA). A secondary antibody (anti-rabbit, dilution 1:5,000; Amersham ECL™ Western Blotting Analysis System; Amersham, UK) was then visualized by the Western blotting enhanced chemiluminescent detection system (ECL; Amersham Biosciences, Amersham, UK). The result was expressed in arbitrary units normalized in relation to β actin, following our previous protocol (Gavrilović et al., 2013).

### Antioxidant enzyme activity

Determination of SODs, CAT, and GPx activity levels was performed using methods previously described by Stojiljković et al. (2009). The activities of SODs, and GPx were determined using appropriate assays by Oxis Bioxytech® (Oxis International, Inc., Portland, OR, USA), while CAT activity was measured by the method of Beutler (1982). SOD assay (Oxis Bioxytech® SOD-525™; Portland, OR, USA) is based on the SOD-mediated increase in the rate of autoxidation of reagent 1 (5,6,6a,11b-tetrahydro-3,9,10-trihydroxybenzo[c] fluorene, R1) in aqueous alkaline solution, yielding a chromophore with maximum absorbance at 525 nm. The change in absorbance at 525 nm was monitored spectrophotometrically. One SOD-525 activity unit was defined as the activity that doubles the autoxidation rate of the control blank. Total SOD activity was measured as described above. Then, the samples were pretreated with ethanol–chloroform reagent (5/3 vol/vol) which inactivates SOD2 and the same procedure for measuring the activity of SOD was performed once again, to determine the activity of SOD1. SOD2 activity was then calculated by subtracting SOD1 activity from total SOD activity (Stojiljković et al., 2009). Determination of CAT activity is based on the rate of H_2_O_2_ degradation by catalase contained in the examined samples. The reaction was performed in an incubation mixture containing 1 M Tris-HCl, 5 mM EDTA, pH 8.0, and monitored spectrophotometrically at 230 nm. One unit of CAT activity was defined as 1 μmol of H_2_O_2_ decomposed per minute under the assay conditions. The assay of GPx activity (Oxis Bioxytech® GPx-340™; Portland, OR, USA) is based on the principle that oxidized glutathione (GSSG) produced upon reduction of an organic peroxide by GPx is immediately recycled to its reduced form (GSH) with concomitant oxidation of NADPH to NADP+. The oxidation of NADPH was monitored spectrophotometrically as a decrease in absorbance at 340 nm. One GPx-340 unit was defined as 1 μmol of NADH oxidized per minute under the assay conditions. The final result for enzyme activity was expressed as units per milligram of protein (U/mg), following our previous protocol (Stojiljković et al., 2009).

### Data analysis

The data are presented as means ± S.E.M. The differences of gene expression (mRNA and protein levels) of SOD1, SOD2, CAT and GPx, as well as the activity of enzymes (SOD1, SOD2, CAT and GPx) between CRS and CRS+Li animals were analyzed by t-test. Statistical analysis was carried out using the SigmaPlot 10.0 with SigmaStat integration. The statistical significance was accepted at *p* < 0.05. Statistical power confirms that the number of animals (*n* = 10) was sufficient for this experiment.

## Results

**Figure 1:**
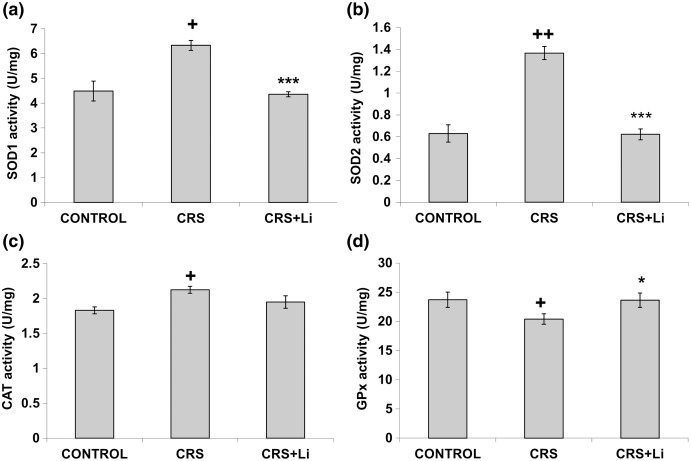
Effects of lithium on the enzyme activities of CuZn superoxide dismutase (SOD1) (A), Mn superoxide dismutase (SOD2) (B), catalase (CAT) (C) and glutathione peroxidase (GPx) (D) in the prefrontal cortex of animals exposed to CRS. The values are means ± S.E.M. of 10 rats. Statistical significance: **+***p* < 0.05 and **++***p* < 0.01 animals exposed to CRS *vs* CONTROL animals; *p < 0.05 and *** *p* < 0.001 animals exposed to CRS+Li *vs* CRS animals (t-test). The final result for enzyme activity was expressed as units per milligram of protein (U/mg).

**Figure 2:**
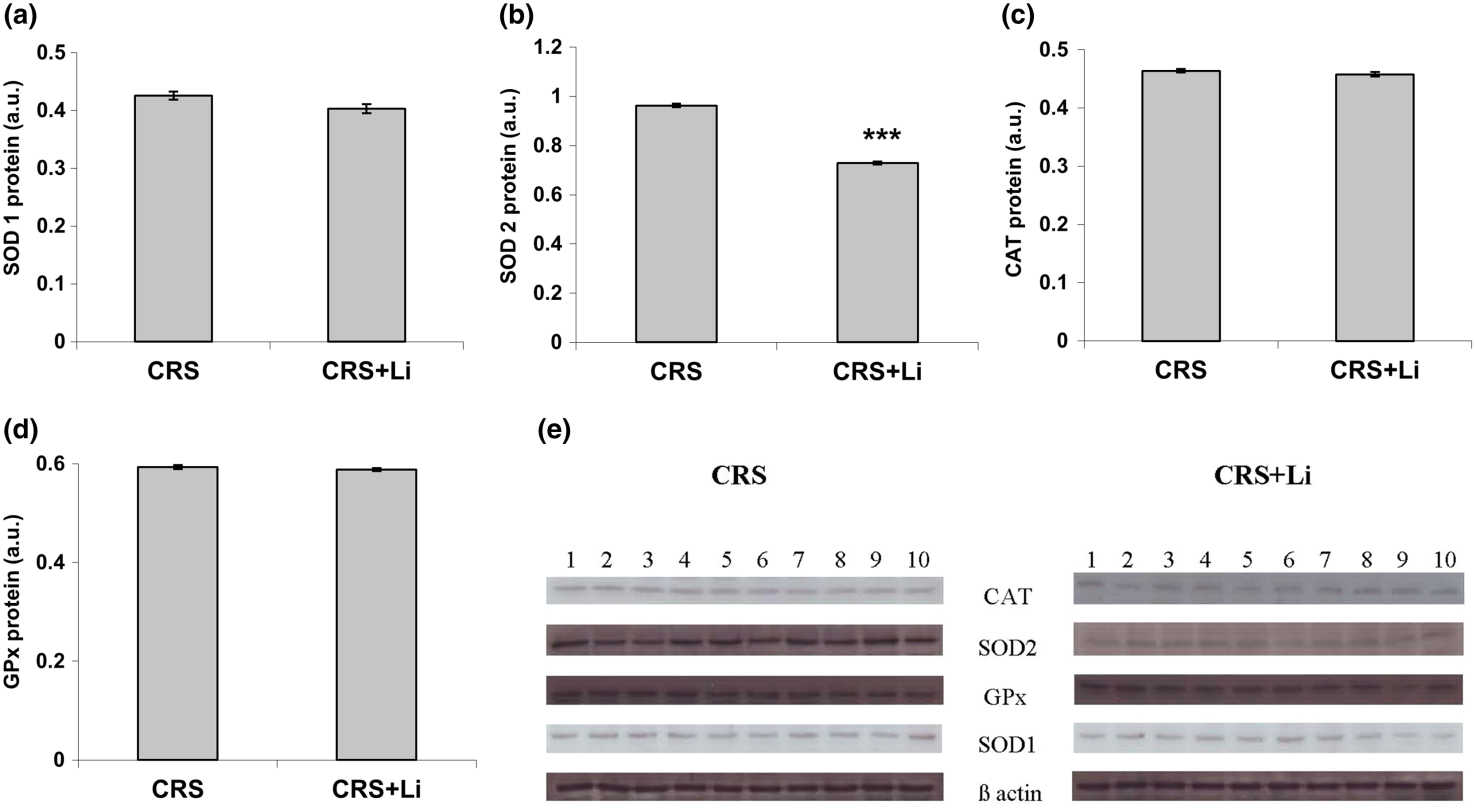
Effects of lithium on the protein levels of CuZn superoxide dismutase (SOD1) (A), Mn superoxide dismutase (SOD2) (B), catalase (CAT) (C) and glutathione peroxidase (GPx) (D) in the prefrontal cortex of animals exposed to CRS. The values are means ± S.E.M. of 10 rats. Statistical significance: ***p < 0.001 animals exposed to CRS+Li *vs* CRS animals (t-test). The final result was expressed in arbitrary units normalized in relation to β actin. (E) Distribution of SOD1, SOD2, CAT, GPx and β-actin proteins in the prefrontal cortex of animals exposed to CRS (*n* = 10), and animals exposed to CRS+Li (*n* = 10).

**Figure 3:**
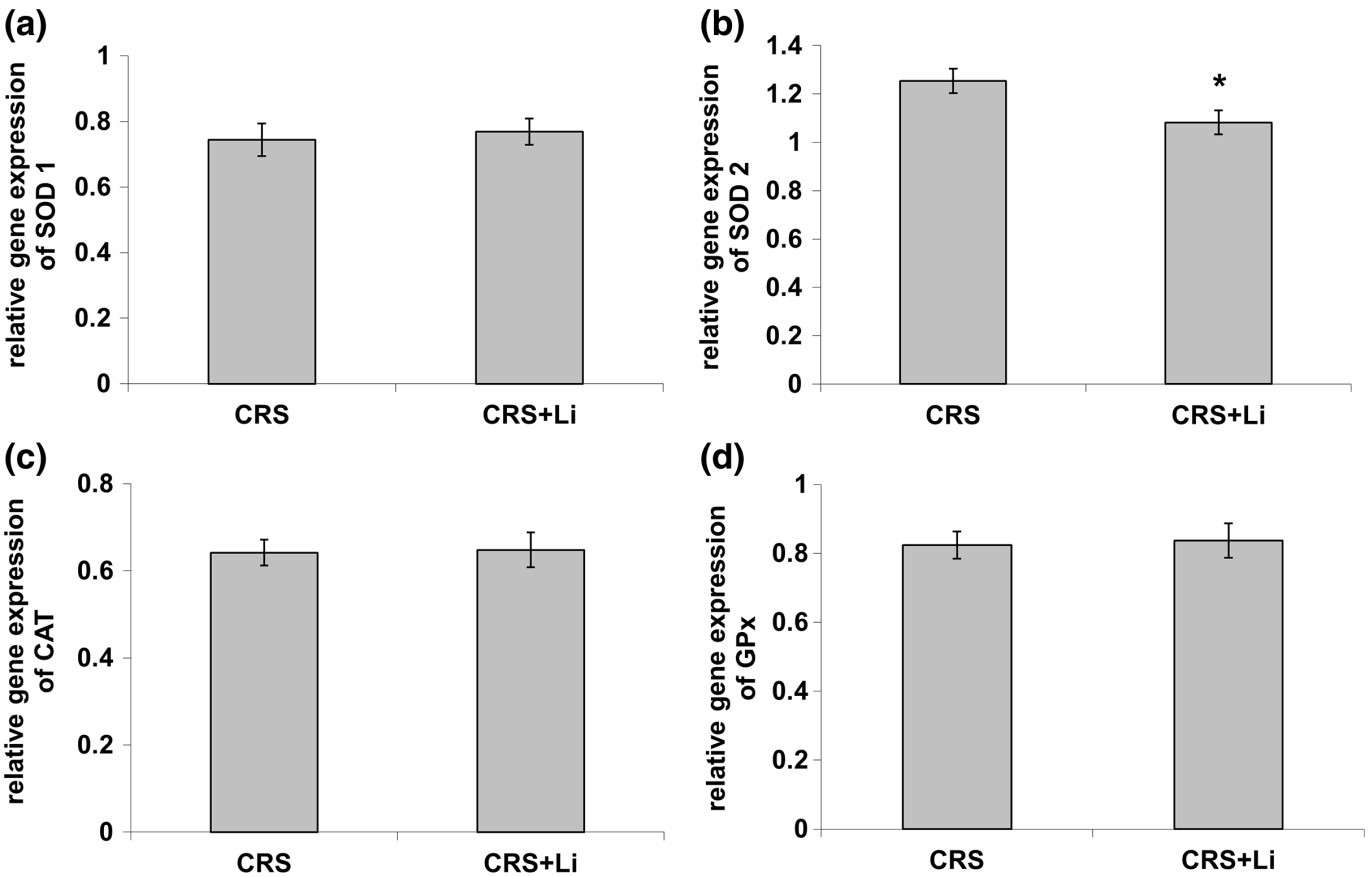
Effects of lithium on mRNA levels of CuZn superoxide dismutase (SOD1) (A), Mn superoxide dismutase (SOD2) (B), catalase (CAT) (C) and glutathione peroxidase (GPx) (D) in the prefrontal cortex of animals exposed to CRS. The values are means ± S.E.M. of 10 rats. Statistical significance: *p < 0.05 animals exposed to CRS+Li *vs* CRS animals (t-test). The final result was expressed as fold change relative to the calibrator and normalized to cyclophyline A.

In our previous study (Popović et al., 2017a) we found that CRS significantly increased SOD1 (*p* < 0.05, t-test, Fig. 1A), SOD2 (*p* < 0.01, t-test, Fig. 1B), and CAT (*p* < 0.05, t-test, Fig. 1C) activity levels in the PFC, compared with CONTROL animals. However, the activity of GPx was decreased (*p* < 0.05, t-test, Fig. 1D) compared to CONTROL animals (Popović et al., 2017a). In this study, we found that lithium treatment in animals exposed to CRS significantly decreased SOD1 activity by 31% (mean = 4.36, t-test, *p* < 0.001, t = 7.290, n = 10, df = 18, Fig. 1A), and SOD2 activity by 54% (mean = 0.622, t-test, *p* < 0.001, t = 9.166, *n* = 10, df = 18, Fig. 1B), as well as the levels of SOD2 protein by 24% (mean = 0.729, t-test, *p* < 0.001, t = 21.979, *n* = 10, df = 18, Fig. 2B), and the levels of SOD2 mRNA by 14% (mean = 1.082, t-test, *p* = 0.024, t = 2.475, *n* = 10, df = 18, Fig. 3B), but significantly increased the activity of GPx by 16% (mean = 23.62, t-test, *p* = 0.047, t = −2.133, df = 18, Fig. 1D) compared with CRS animals.

However, lithium treatment did not change the gene expression (protein and mRNA levels) of SOD1 (Figs. 2A and 3A), as well as the gene expression of GPx (Figs. 2D and 3D) in CRS animals. Also, lithium treatment in animals exposed to CRS did not change the enzyme activity and the gene expression of CAT (protein and mRNA levels) (Figs. 1C, 2C and 3C) compared with CRS animals.

In addition, CRS animals treated with lithium showed a decreased ratio of SOD1/CAT and SOD2/CAT compared with CRS animals.

## Discussion

In our previous study we confirmed that CRS influenced depressive-like behavior in rats (Popović et al., 2017b), increased the activity of prefrontal SOD1, SOD2 and CAT, and decreased GPx activity (Popović et al., 2017a). In addition, our early finding confirms that modulated activities of antioxidative enzymes SOD, CAT and GPx could represent the markers of oxidative stress (Popović et al., 2019). It is known that the nuclear factor 2-related factor 2 Nrf2 pathway participates in the development and progression of oxidative stress injury (Chen et al., 2015; Cui et al., 2015; Zhu et al., 2016). Under normal conditions, Nrf2 is present in the cytoplasm in combination with Kelch-like ECH-related protein 1 (Keap1) (Tkachev, Menshchikova & Zenkov, 2011). The Nrf-2/Keap-1 signaling pathway provides cells with a defense mechanism against oxidative stress by regulating the expression of enzymes that have key roles in the anti-oxidative stress response and detoxification (Jaramillo & Zhang, 2013). It was already published that the lithium treatment inhibited hydrogen peroxide-induced cell death in primary cultured rat cerebral cortical cells. This further corroborates that lithium is involved in the protection against oxidative stress-induced cell death (Cui et al., 2007). Moreover, our previous findings that lithium decreased hippocampal MDA levels in chronically stressed rats (Popović et al., 2019) unequivocally support the conclusion that lithium contributes to the reduction of oxidative stress in CRS conditions. Also, our previous finding confirms that lithium stabilized the behavior of animals with depressive-like behavior and made them better prepared for a new challenge (Gavrilović et al., 2021). Several studies have confirmed that the lithium treatment changed mRNA expression of Nrf2 (Milani et al., 2013; Gan & Johnson, 2014; Dodson et al., 2015; Alural et al., 2015). This study’s findings that the SODs and GPx activities in chronically stressed rats returned to the levels of unstressed animals corroborate that lithium is involved in maintaining the stability of prefrontal antioxidant defense system. It is possible that lithium regulates redox balance in animals exposed to CRS *via* several anti-oxidative proteins, including Nrf-2 and Keap-1. Furthermore, the current study demonstrated that lithium treatment did not alter the enzyme activity of prefrontal CAT in animals exposed to CRS. The literature data confirm that elevated SOD/CAT ratio suggests an increase in oxidative stress levels, mostly associated with elevation in cell hydrogen peroxide concentration (Gsell et al., 1995). Our finding confirms that the reduction in SOD/CAT ratio may indicate lower oxidative stress in the PFC of chronically stressed rats after lithium treatment, which is in line with our previous findings (Popović et al., 2019). Based on our results, it could be speculated that lithium maintains the stability of prefrontal antioxidant defense system by orchestrating the change of SODs and GPx activities in chronically stressed animals.

## Conclusions

In the present study we found that in animals exposed to CRS, the lithium treatment significantly decreased the activity of prefrontal SOD1 and SOD2, but significantly increased the activities of GPx to the levels of unstressed animals found in our previous research. Modulation of the prefrontal enzyme activities of SOD1, SOD2, and GPx in chronically stressed animals treated with lithium may by a pivotal step in fully understanding the role of lithium in maintaining the stability of prefrontal antioxidant defense system in neuropsychiatric disorders caused by chronic stress. The knowledge of the lithium effects on redox balance regulation will guide the development of novel therapeutic strategies, aiming at reduction of oxidative stress in pathological conditions.

## Supplemental Information

10.7717/peerj.13020/supp-1 Supplemental Information 1 Raw Data.

10.7717/peerj.13020/supp-2 Supplemental Information 2 Photos of Blots.

10.7717/peerj.13020/supp-3 Supplemental Information 3 Author Checklist.

## Additional Information and Declarations

### Data Availability

The following information was supplied regarding data availability: The raw data is available in the Supplemental Files.

## References

[ref-1] Alural B, Ozerdem A, Allmer J, Genc K, Genc S (2015). Lithium protects against paraquat neurotoxicity by NRF2 activation and miR-34a inhibition in SH-SY5Y cells. Frontiers in Cellular Neuroscience.

[ref-2] Andreazza AC, Cassini C, Rosa AR, Leite MC, de Almeida LMV, Nardin P, Cunha ABN, Ceresér KM, Santin A, Gottfried C, Salvador M, Kapczinski F, Gonçalves CA (2007). Serum S100B and antioxidant enzymes in bipolar patients. Journal of Psychiatric Research.

[ref-3] Arnsten AFT (2009). Stress signalling pathways that impair prefrontal cortex structure and function. Nature Reviews Neuroscience.

[ref-4] Beutler E (1982). Catalase. Red cell metabolism. A Manual of Biochemical Methods.

[ref-5] Bhatt S, Nagappa AN, Patil CR (2020). Role of oxidative stress in depression. Drug Discovery Today.

[ref-6] Chen F, Zhang N, Ma X, Huang T, Shao Y, Wu C, Wang Q (2015). Naringin alleviates diabetic kidney disease through inhibiting oxidative stress and inflammatory reaction. PLOS ONE.

[ref-7] Cui G, Luk SC, Li RA, Chan KK, Lei SW, Wang L, Shen H, Leung GP, Lee SM (2015). Cytoprotection of baicalein against oxidative stress-induced cardiomyocytes injury through the Nrf2/Keap1 pathway. Journal of Cardiovascular Pharmacology.

[ref-8] Cui J, Shao L, Young LT, Wang JF (2007). Role of glutathione in neuroprotective effects of mood stabilizing drugs lithium and valproate. Neuroscience.

[ref-9] Deutch AY (1993). Prefrontal cortical dopamine systems and the elaboration of functional corticostriatal circuits: implications for schizophrenia and Parkinson’s disease. Journal of Neural Transmission.

[ref-10] Dodson M, Redmann M, Rajasekaran NS, Darley-Usmar V, Zhang J (2015). KEAP1-NRF2 signalling and autophagy in protection against oxidative and reductive proteotoxicity. Biochemical Journal.

[ref-11] Fibiger HC (1995). Neurobiology of depression: focus on dopamine. Advances in Biochemical Psychopharmacology.

[ref-12] Gamaro GD, Michalowski MB, Catelli DH, Xavier MH, Dalmaz C (1999). Effect of repeated restraint stress on memory in different tasks. Brazilian Journal of Medical and Biological Research.

[ref-13] Gan L, Johnson JA (2014). Oxidative damage and the Nrf2-ARE pathway in neurodegenerative diseases. Biochimica et Biophysica Acta.

[ref-14] Gavrilović L, Popović N, Stojiljković V, Pejić S, Todorović A, Pavlović I, Pantelić M, Pajović SB (2021). Effects of mood stabilizer lithium on noradrenergic turnover in the prefrontal cortex of chronically stressed rats. Neuroendocrinology Letters.

[ref-15] Gavrilovic L, Spasojevic N, Dronjak S (2010). Subsequent stress increases gene expression of catecholamine synthetic enzymes in cardiac ventricles of chronic-stressed rats. Endocrine.

[ref-16] Gavrilović L, Stojiljković V, Kasapović J, Pejić S, Todorović A, Pajović SB, Dronjak S (2012). Forced exercise changes catecholamine synthesis in the spleen of adult rats. Journal of Neuroimmunology.

[ref-17] Gavrilović L, Stojiljković V, Kasapović J, Popović N, Pajović SB, Dronjak S (2013). Treadmill exercise does not change gene expression of adrenal catecholamine biosynthetic enzymes in chronically stressed rats. Anais da Academia Brasileira de Ciencias.

[ref-18] Glahn DC, Bearden CE, Cakir S, Barrett JA, Najt P, Serap Monkul E, Maples N, Velligan DI, Soares JC (2006). Differential working memory impairment in bipolar disorder and schizophrenia: effects of lifetime history of psychosis. Bipolar Disorders.

[ref-19] Goldman-Rakic PS (1994). Working memory dysfunction in schizophrenia. Journal of Neuropsychiatry and Clinical Neurosciences.

[ref-20] Goldman-Rakic PS (1995). Cellular basis of working memory. Neuron.

[ref-21] Gsell W, Conrad R, Hickethier M, Sofic E, Frölich L, Wichart I, Jellinger K, Moll G, Ransmayr G, Beckmann H (1995). Decreased catalase activity but unchanged superoxide dismutase activity in brains of patients with dementia of Alzheimer type. Journal of Neurochemistry.

[ref-22] Huang RR, Hu W, Yin YY, Wang YC, Li WP, Li WZ (2014). Chronic restraint stress promotes learning and memory impairment due to enhanced neuronal endoplasmic reticulum stress in the frontal cortex and hippocampus in male mice. International Journal of Molecular Medicine.

[ref-23] Jaramillo MC, Zhang DD (2013). The emerging role of the Nrf2-Keap1 signaling pathway in cancer. Genes & Development.

[ref-24] Kim KS, Han PL (2006). Optimization of chronic stress paradigms using anxiety- and depression-like behavioral parameters. Journal of Neuroscience Research.

[ref-25] Machado-Vieira R, Manji HK, Zarate CA (2009). The role of lithium in the treatment of bipolar disorder: convergent evidence for neurotrophic effects as a unifying hypothesis. Bipolar Disorders.

[ref-26] Mattes JA (1980). The role of frontal lobe dysfunction in childhood hyperkinetics. Comprehensive Psychiatry.

[ref-27] Mazure CM, Spiegel D (1995). Does stress cause psychiatric illness?. Progress in Psychiatry.

[ref-28] Milani P, Ambrosi G, Gammoh O, Blandini F, Cereda C (2013). SOD1 and DJ-1convergeat Nrf2 pathway: aclue for antioxid ant therapeutic potential in neurodegeneration. Oxidative Medicine and Cellular Longevity.

[ref-29] Nonaka S, Chuang DM (1998). Neuroprotective effects of chronic lithium on focal cerebral ischemia in rats. NeuroReport.

[ref-30] Popović N, Pajović SB, Stojiljković V, Pejić S, Todorović A, Pavlović I, Gavrilović L (2017a). Prefrontal catecholaminergic turnover and antioxidant defense system of chronically stressed rats. Folia Biologica-Kraków.

[ref-31] Popović N, Pajović SB, Stojiljković V, Todorović A, Pejić S, Pavlović I, Gavrilović L (2017b). Relationship between behaviors and catecholamine content in prefrontal cortex and hippocampus of chronically stressed rats.

[ref-32] Popović N, Stojiljković V, Pejić S, Todorović A, Pavlović I, Gavrilović L, Pajović SB (2019). Modulation of hippocampal antioxidant defense system in chronically stressed rats by lithium. Oxidative Medicine and Cellular Longevity.

[ref-33] Radley JJ, Sisti HM, Hao J, Rocher AB, Mccall T, Hof PR, Mcewen BS, Morrison JH (2004). Chronic behavioral stress induces apical dendritic reorganization in pyramidal neurons of the medial prefrontal cortex. Neuroscience.

[ref-34] Schwab RS, Zieper I (1965). Effects of mood, motivation, stress, and alertness on the performance in Parkinson’s disease. Psychiatry Neurology.

[ref-35] Shao L, Young LT, Wang JF (2005). Chronic treatment with mood stabilizers lithium and valproate prevents excitotoxicity by inhibiting oxidative stress in rat cerebral cortical cells. Biological Psychiatry.

[ref-36] Shin LM, Rauch SL, Pitman RK (2006). Amygdala, medial prefrontal cortex, and hippocampal function in PTSD. Annals of the New York Academy of Sciences.

[ref-37] Stich TM (1990). Determination of protein covalently bound to agarose supports using bicinchoninic acid. Annals of Biochemistry.

[ref-38] Stojiljković V, Todorović A, Pejić S, Kasapović J, Saičić ZS, Radlović N, Pajović SB (2009). Antioxidant status and lipid peroxidation in small intestinal mucosa of children with celiac disease. Clinical Biochemistry.

[ref-39] Tkachev VO, Menshchikova EB, Zenkov NK (2011). Mechanism of the Nrf2/Keap1/ARE signaling system. Biochemistry.

[ref-40] Wang Y, Kan H, Yin Y, Wu W, Hu W, Wang M, Li W (2014). Protective effects of ginsenoside Rg1 on chronic restraint stress induced learning and memory impairments in male mice. Pharmacology Biochemistry and Behavior.

[ref-41] Weinberger DR, Berman KF, Zec RF (1986). Physiologic dysfunction of dorsolateral prefrontal cortex in schizophrenia, I: regional cerebral blood flow evidence. Archives of General Psychiatry.

[ref-42] Wellman CL (2001). Dendritic reorganization in pyramidal neurons in medial prefrontal cortex after chronic corticosterone administration. Journal of Neurobiology.

[ref-43] Wood GE, Young LT, Reagan LP, Chen B, McEwen BS (2004). Stress-induced structural remodeling in hippocampus: prevention by lithium treatment. Proceedings of the National Academy of Sciences of the United States of America.

[ref-44] Zhu Y, Zhang YJ, Liu WW, Shi AW, Gu N (2016). Salidroside suppresses HUVECs cell injury induced by oxidative stress through activating the Nrf2 signaling pathway. Molecules.

