# Supplementary figures and images for: Antioxidant defense system in the prefrontal cortex of chronically stressed rats treated with lithium
Source: PeerJ. 2022 Mar 23;10:e13020. doi: 10.7717/peerj.13020 (PMC8957266; doi:10.7717/peerj.13020)

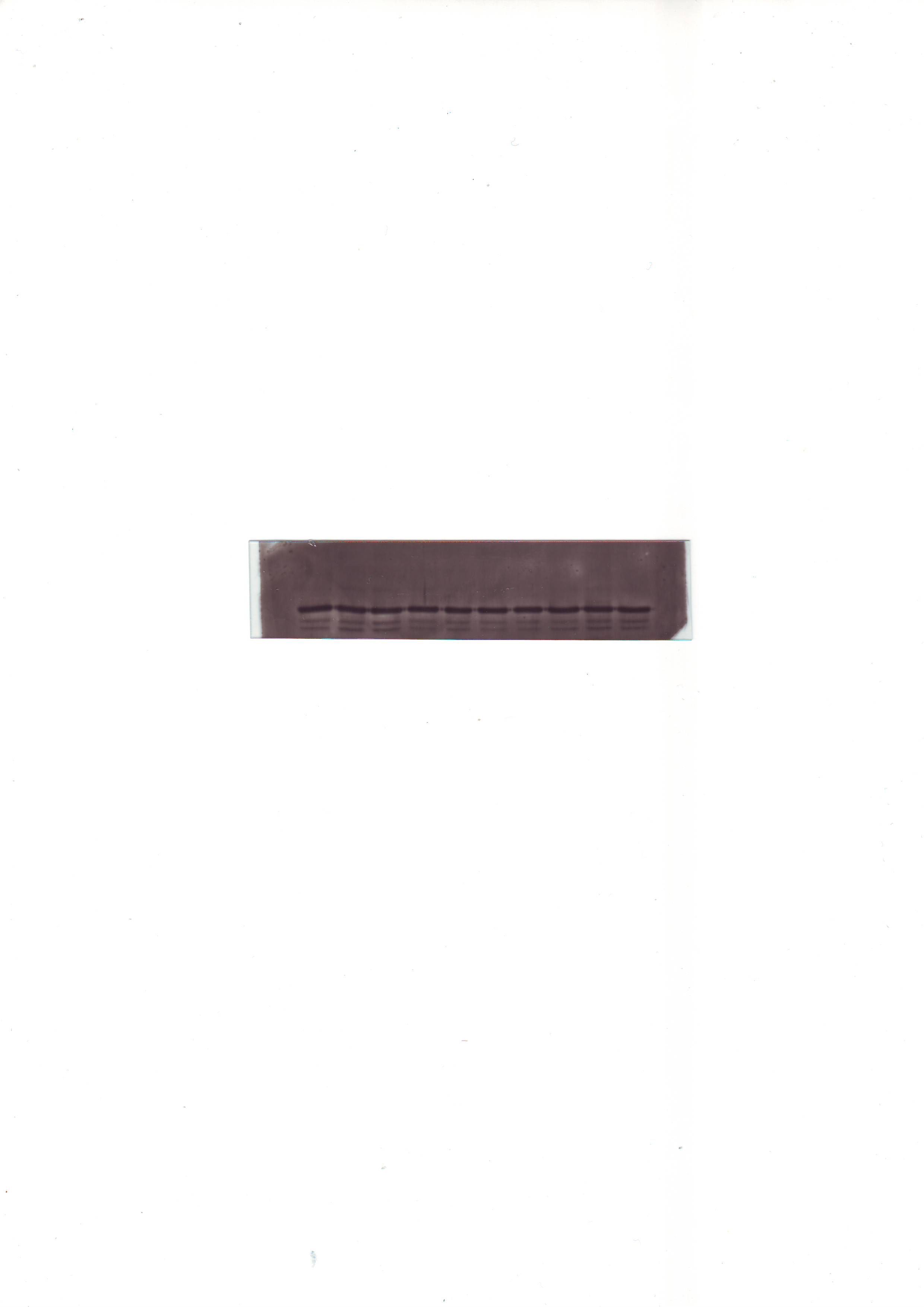

Supplement: Supplemental Information 2 [file peerj-10-13020-s002.zip › Photos of Blots/B actin CRS.JPG]

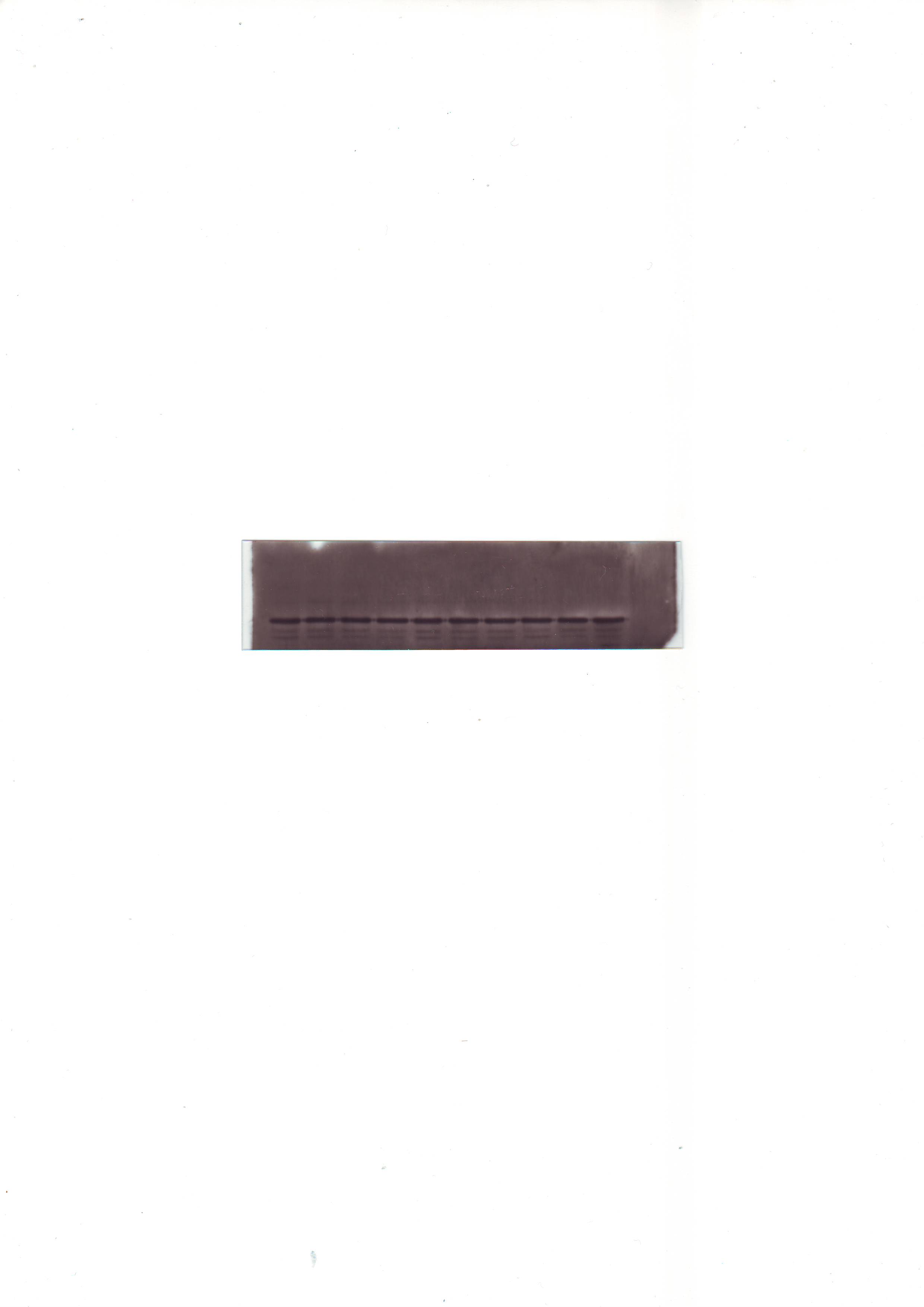

Supplement: Supplemental Information 2 [file peerj-10-13020-s002.zip › Photos of Blots/B actin CRS+Li.JPG]

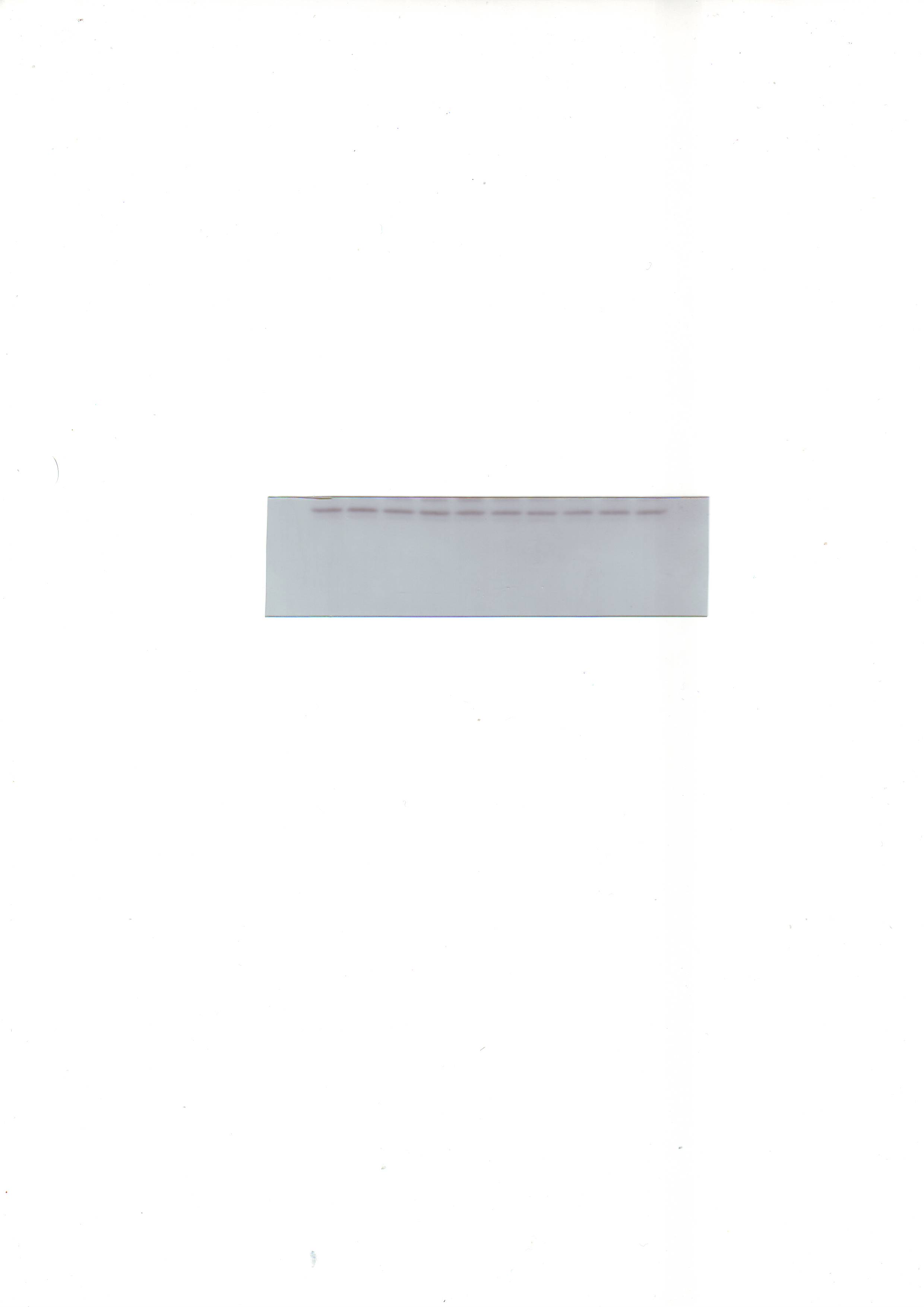

Supplement: Supplemental Information 2 [file peerj-10-13020-s002.zip › Photos of Blots/CAT CRS doc.JPG]

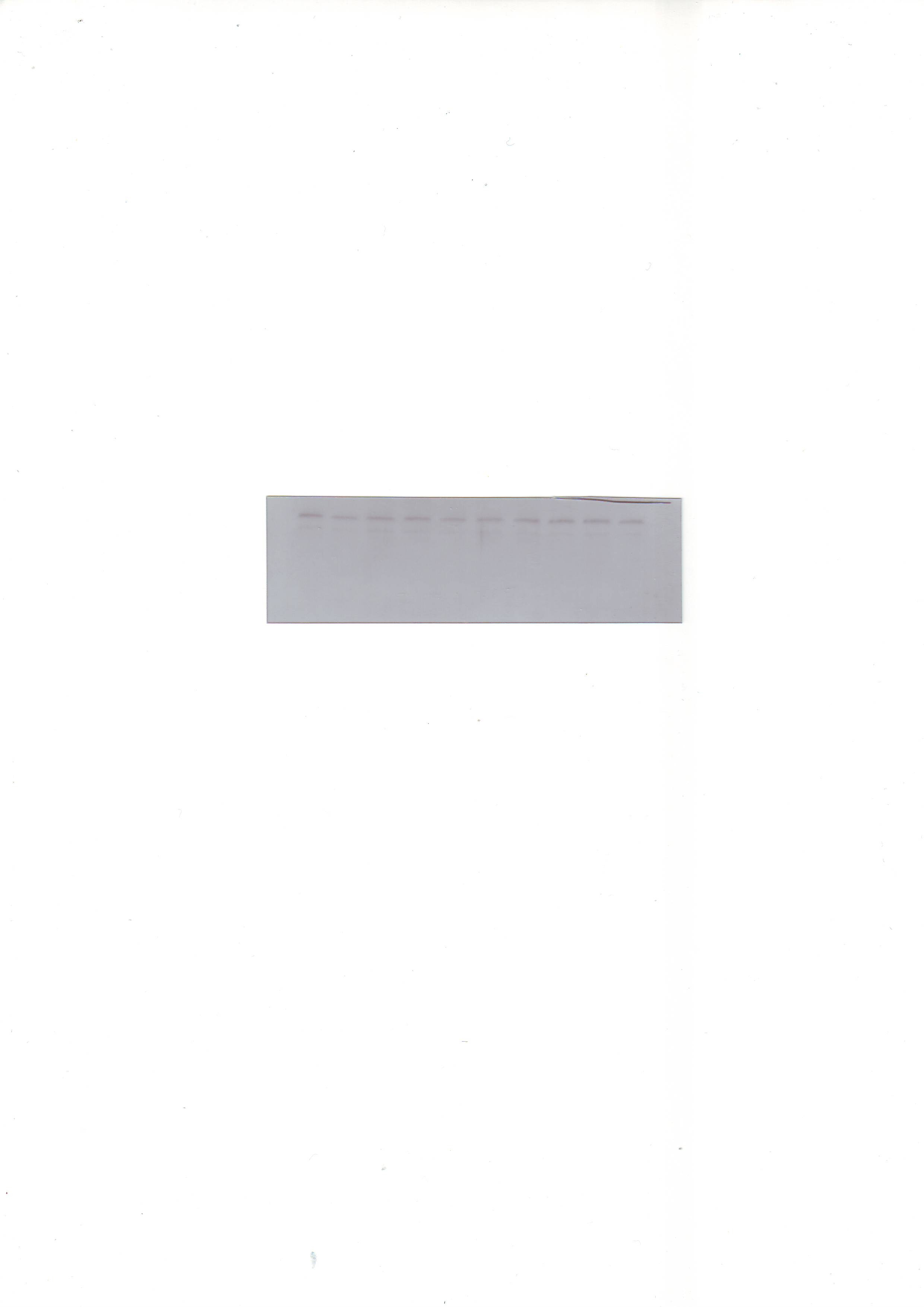

Supplement: Supplemental Information 2 [file peerj-10-13020-s002.zip › Photos of Blots/CAT CRS+LI.jpg]

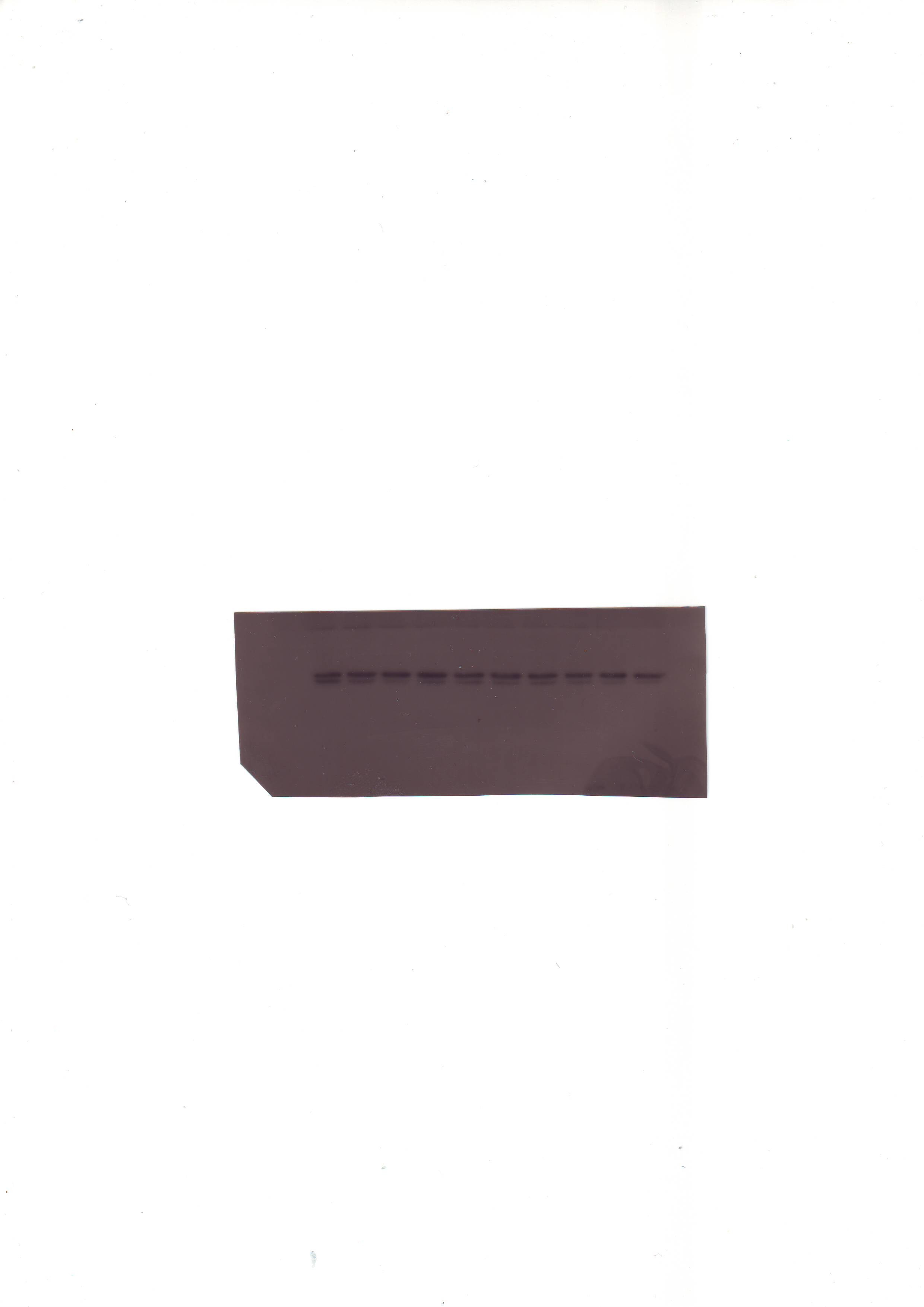

Supplement: Supplemental Information 2 [file peerj-10-13020-s002.zip › Photos of Blots/GPx CRS doc.jpg]

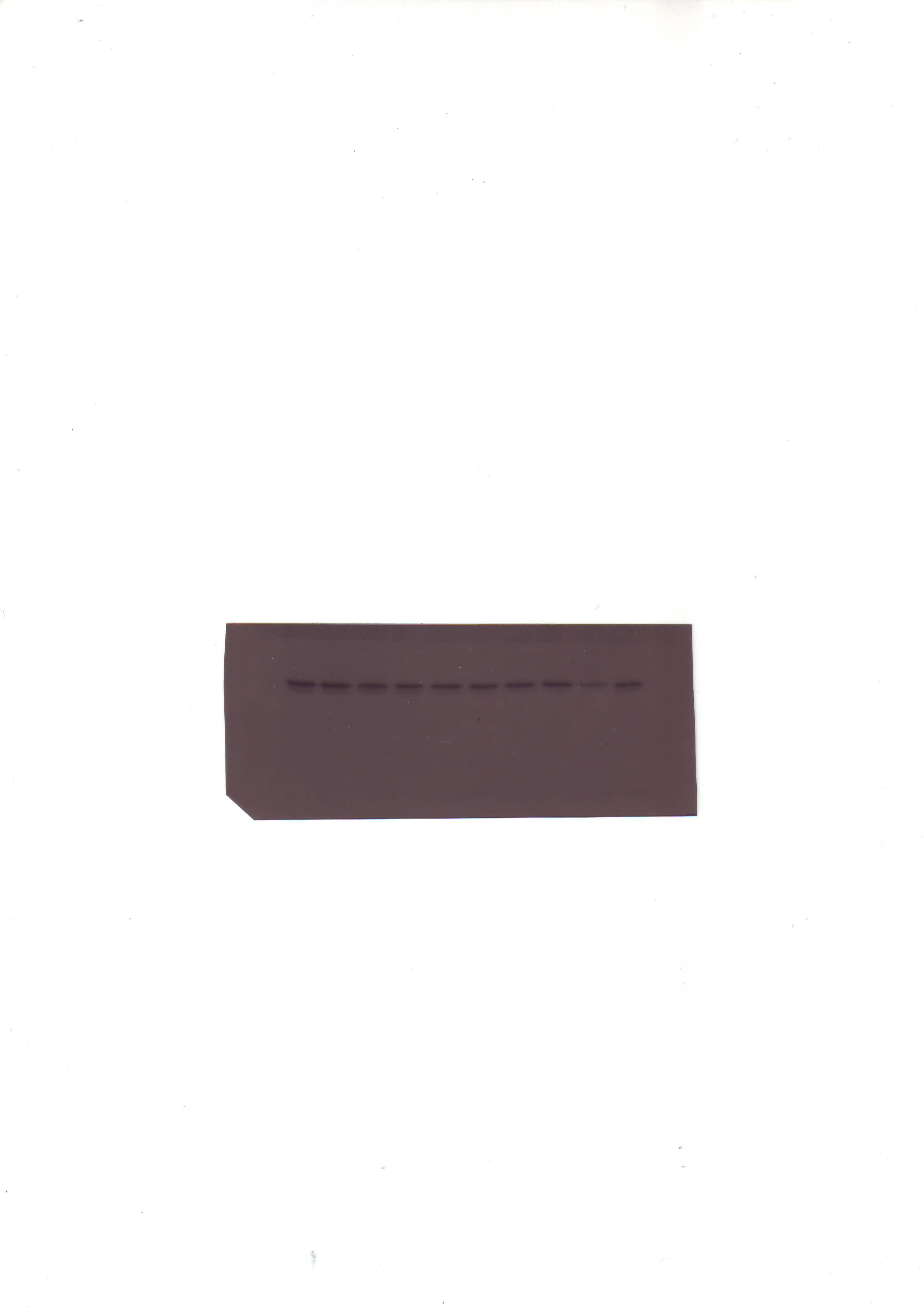

Supplement: Supplemental Information 2 [file peerj-10-13020-s002.zip › Photos of Blots/GPx+Li CRS doc.JPG]

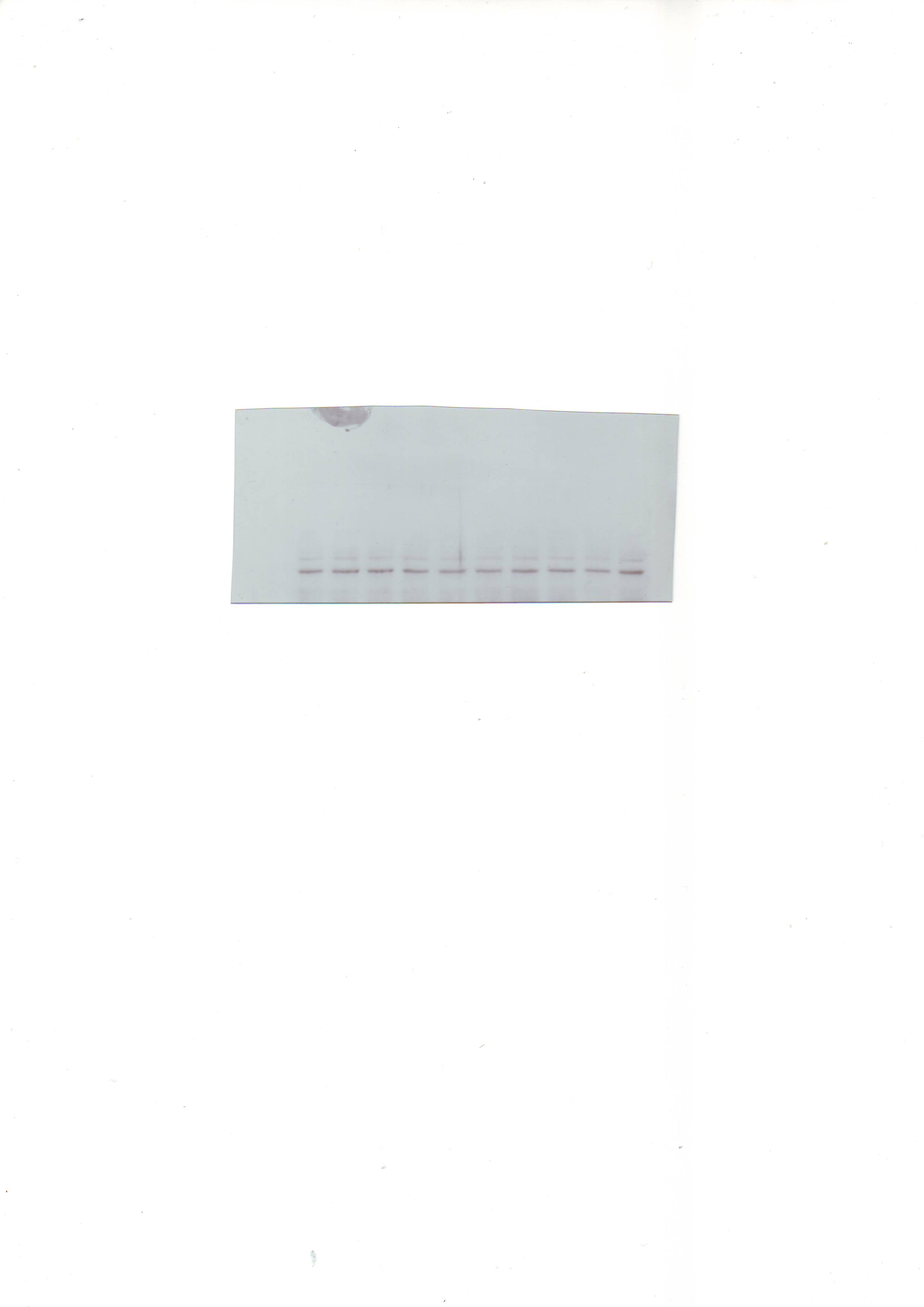

Supplement: Supplemental Information 2 [file peerj-10-13020-s002.zip › Photos of Blots/SOD1 CRS doc.JPG]

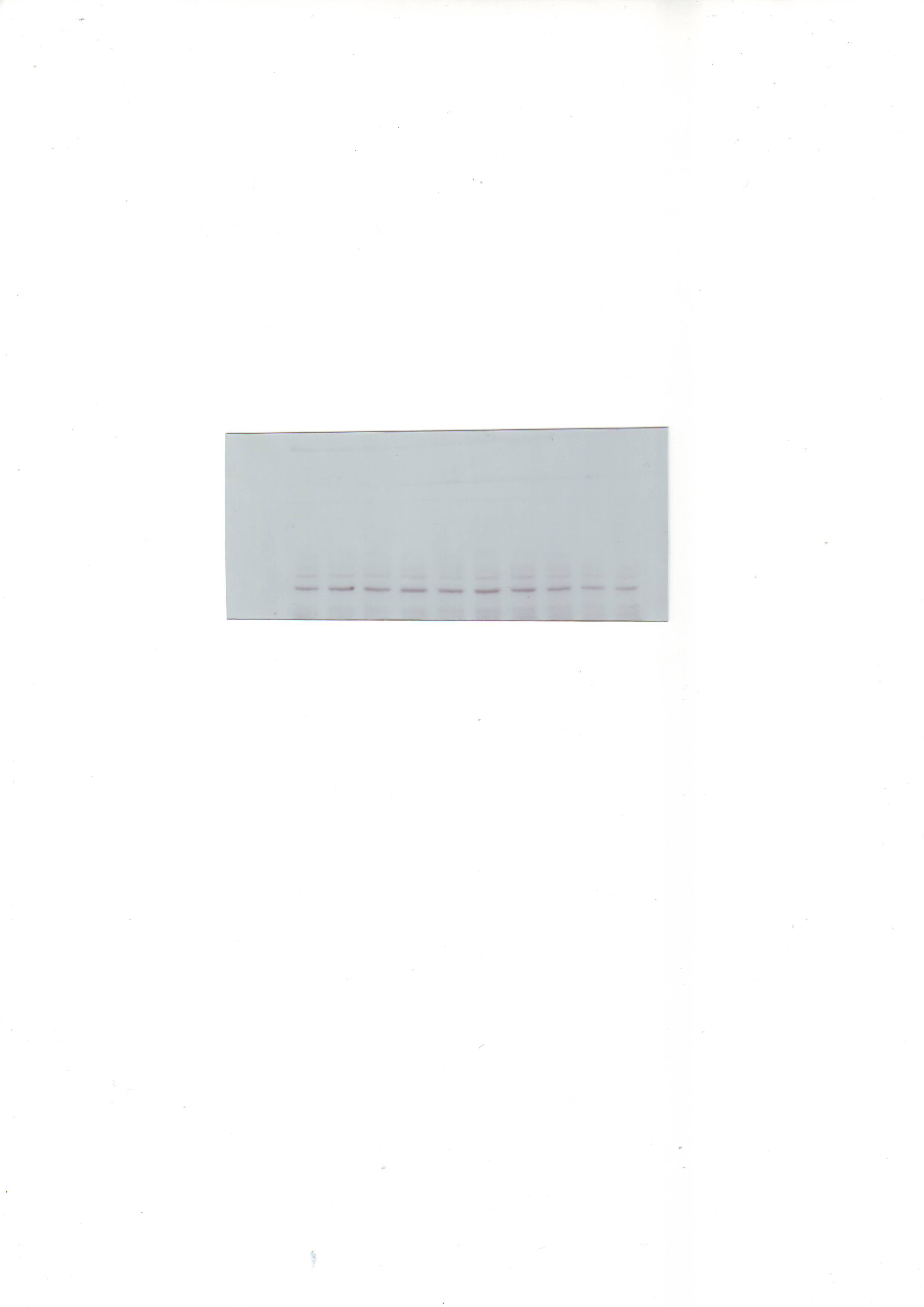

Supplement: Supplemental Information 2 [file peerj-10-13020-s002.zip › Photos of Blots/SOD1 CRS+Li doc.JPG]

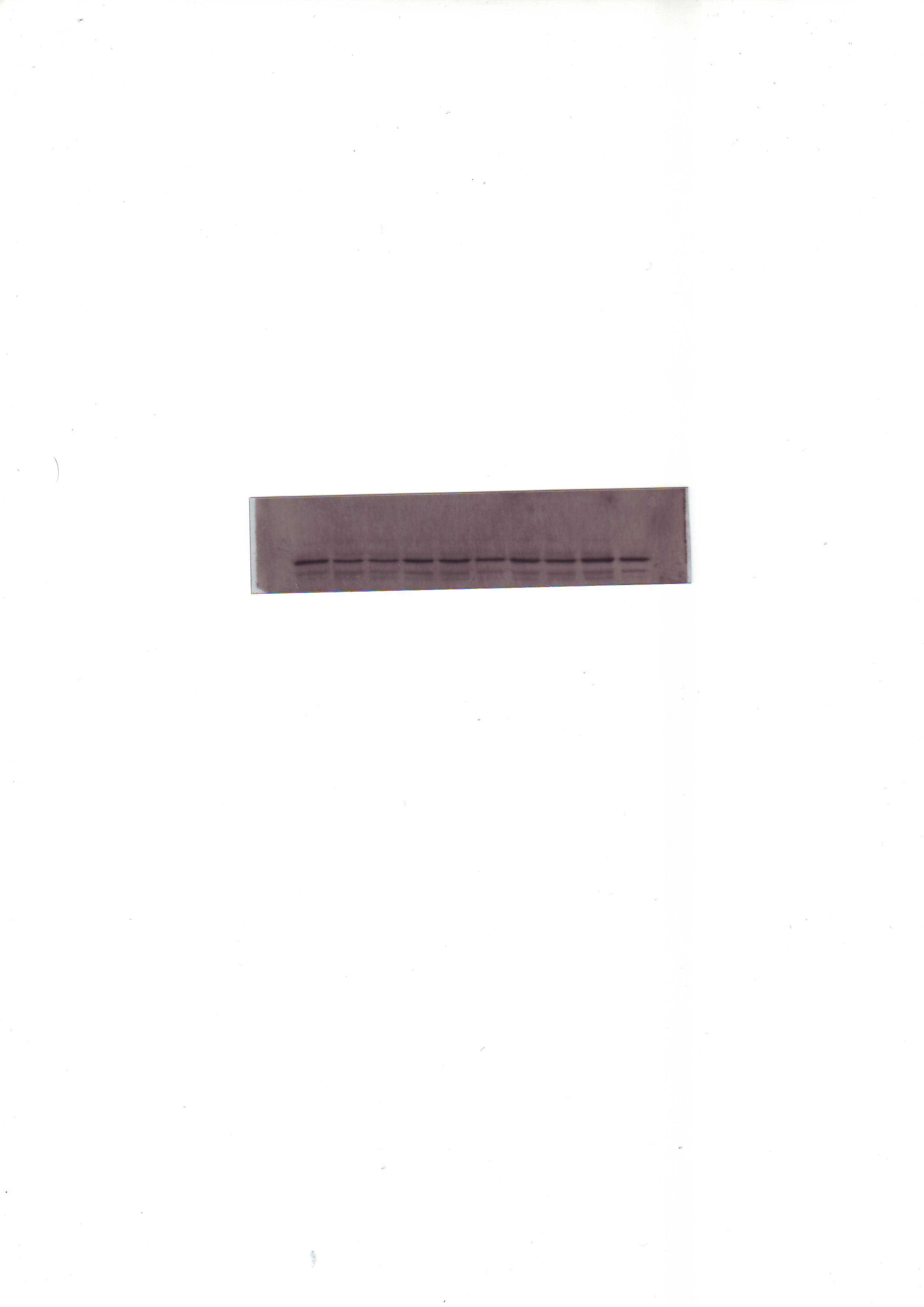

Supplement: Supplemental Information 2 [file peerj-10-13020-s002.zip › Photos of Blots/SOD2 CRS doc.JPG]

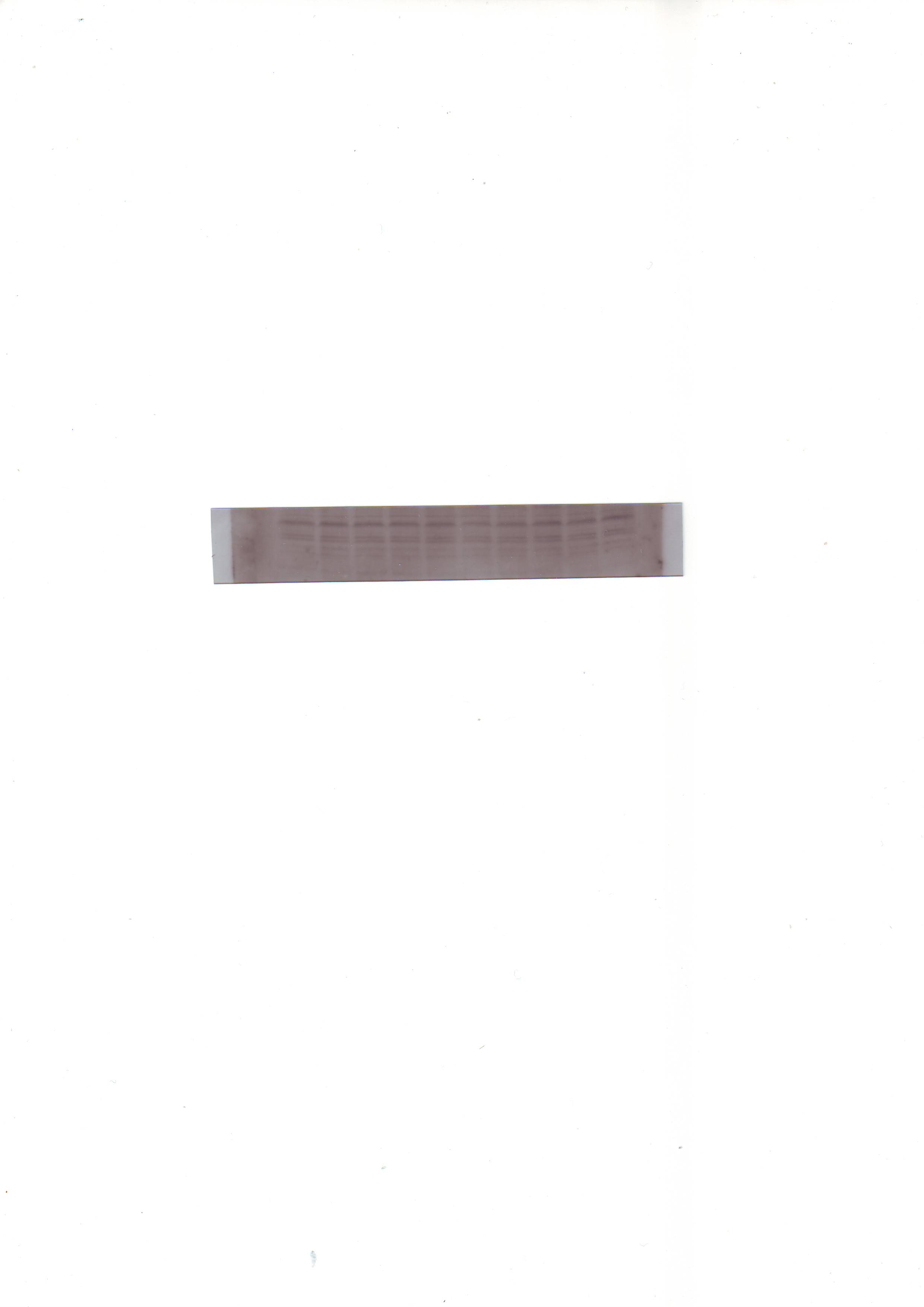

Supplement: Supplemental Information 2 [file peerj-10-13020-s002.zip › Photos of Blots/SOD2 CRS+Li doc.JPG]
